# KrillDB: A *de novo* transcriptome database for the Antarctic krill (*Euphausia superba*)

**DOI:** 10.1371/journal.pone.0171908

**Published:** 2017-02-10

**Authors:** Gabriele Sales, Bruce E. Deagle, Enrica Calura, Paolo Martini, Alberto Biscontin, Cristiano De Pittà, So Kawaguchi, Chiara Romualdi, Bettina Meyer, Rodolfo Costa, Simon Jarman

**Affiliations:** 1 Department of Biology, University of Padova, Padova, Italy; 2 Australian Antarctic Division, Kingston, Tasmania, Australia; 3 Alfred Wegener Institute, Helmholtz Centre for Polar and Marine Research, Bremerhaven, Germany; 4 Institute for Chemistry and Biology of the Marine Environment, Carl von Ossietzky University Oldenburg, Oldenburg, Germany; University of Ferrara, ITALY

## Abstract

Antarctic krill (*Euphausia superba*) is a key species in the Southern Ocean with an estimated biomass between 100 and 500 million tonnes. Changes in krill population viability would have catastrophic effect on the Antarctic ecosystem. One looming threat due to elevated levels of anthropogenic atmospheric carbon dioxide (CO_2_) is ocean acidification (lowering of sea water pH by CO_2_ dissolving into the oceans). The genetics of Antarctic krill has long been of scientific interest for both for the analysis of population structure and analysis of functional genetics. However, the genetic resources available for the species are relatively modest. We have developed the most advanced genetic database on *Euphausia superba*, KrillDB, which includes comprehensive data sets of former and present transcriptome projects. In particular, we have built a *de novo* transcriptome assembly using more than 360 million Illumina sequence reads generated from larval krill including individuals subjected to different CO_2_ levels. The database gives access to: 1) the full list of assembled genes and transcripts; 2) their level of similarity to transcripts and proteins from other species; 3) the predicted protein domains contained within each transcript; 4) their predicted GO terms; 5) the level of expression of each transcript in the different larval stages and CO_2_ treatments. All references to external entities (sequences, domains, GO terms) are equipped with a link to the appropriate source database. Moreover, the software implements a full-text search engine that makes it possible to submit free-form queries. KrillDB represents the first large-scale attempt at classifying and annotating the full krill transcriptome. For this reason, we believe it will constitute a cornerstone of future approaches devoted to physiological and molecular study of this key species in the Southern Ocean food web.

## Introduction

Antarctic krill (*Euphausia superba*) is a key species in the Southern Ocean with an estimated biomass between 100 and 500 million tons. The species provides a critical ecological link between primary producers and apex predators and supports commercial fisheries [1].

Changes in krill population viability would have catastrophic effect on the Antarctic ecosystem [1,2]. One looming threat is ocean acidification (OA)–this is lowering of sea water pH due to elevated levels of anthropogenic atmospheric carbon dioxide (CO_2_) dissolving into the oceans [3]. This threat is recognized to be a global issue for the marine environment, but cold southern ocean waters are particularly susceptible due to high solubility of CO_2_ [4]. Recent experimental work has documented the impact of increased CO_2_ levels on krill development [2,5,6].

There has long been interest in the ecological genetics of Antarctic krill both for the analysis of population structure [7,8] and analysis of functional genetics [9–12]. However, the genetic resources available for this species are relatively modest. The genome size of the species is exceptionally large [13]; at 48.53 pg, it is more than 15 times larger than the human genome. Arthropods are the most diverse animal phylum, but their genomic resources have a relatively narrow taxonomic focus and little insight into the krill genome can be obtained from related species [14]. Even with rapid advance in DNA sequencing technology the current prospects for carrying out a complete genome sequencing and assembly project on krill is poor [15]. Sequencing of the krill transcriptome is much more feasible and could provide valuable information on coding sequences of many genes and will expedite the development of gene-linked markers [16].

Some transcriptome resources have been developed for this krill species including: sequencing of a few thousand ESTs [9,10], 454 assemblies from hundreds of thousands of sequence reads [11,17] and [18] (only raw reads available) and development of a 32,217 probe microarray [19]. However, all these resources are not yet available to the community as an organized resource as a database.

We have developed the most advanced genetic database on *Euphausia superba*, which include comprehensive data sets of former and present transcriptome projects. In particular, we have built a *de novo* transcriptome assembly using more than 360 million Illumina sequence reads generated from larval krill exposed to either normal or elevated CO_2_ levels (short-term exposure employed to increase the representation of alternative transcripts, in particular with respect to an environmental stress). Using a meta-assembly strategy, we have produced and annotated a comprehensive Antarctic krill transcriptome that will contribute to the ongoing development of genomic resources for this species.

Here we present KrillDB, a web-based graphical interface to our annotation results. The database gives access to the full list of assembled genes and transcripts. This includes their reconstructed sequences, their level of similarity to transcripts and proteins from other species, the predicted protein domains contained within each transcript, the GO terms that can be linked to each transcript (based on previously described gene product characteristics) and the level of expression of each transcript in normal conditions and at the different CO_2_ concentration levels. The KrillDB software implements a full-text search engine that makes it possible to quickly find all krill genes/transcripts linked to a query by submitting any free-form search text.

## Material and methods

### RNA-seq data

#### Krill collection and larval krill CO_2_ exposure

Adult krill were collected in April 2011 from the Indian Ocean sector of the Southern Ocean (64°09' S, 100°460 E) and were maintained in the Australian Antarctic Division's marine research aquarium (see [20] for details). Krill were collected using permit AMLR 08-12-2337, issued under "Commonwealth of Australia, Antarctic Marine Living Resources Act 1981”. These krill matured naturally in the laboratory, and gravid females spawned between December 2011 and February 2012. After hatching batches of larvae were reared through the 12 recognizable stages of development over several months [21]. Larvae at two different stages of development were used in our experiment: (a) Stage 4 (*Calyptopis I*) reached at approximately 2 weeks post-hatching and (b) Stage 11 (*Furcilia V*), approximately 16 weeks post-hatching. The procedure used for rearing these larvae is described in [20].

Larvae used in the experiment were reared under common ambient pCO_2_ conditions at 0.5°C and were randomly assigned to a *p*CO_2_ level treatment (control, 1000 or 2000 μatm *p*CO_2_). A total of six experimental jars (2 larval stages x 3 *p*CO_2_ levels) were used. For *Calyptopis I*, 25 individuals were randomly selected and placed in each jar. Due to their larger size, only 5 *Furcilia V* larvae were used in each jar. The CO_2_ exposure experiment lasted 48 hours and was carried out concurrently for both larval stages in March 2012. Details of the experimental set-up are described in [5]. Briefly, sea water from a header tank (70 l at 0.5°C) was equilibrated with air (control) or CO_2_-enriched air before being delivered to experimental jars (250 ml clear polycarbonate) containing krill larvae. The pCO_2_ levels of CO_2_-enriched air were monitored directly and also indirectly by pH measurement of the sea water. Experimental jars for each level of pCO_2_ were kept on separate shelves of a refrigerator maintained at 0.5°C. Overflow effluent from each jar was drained into a sump, and recirculated through a degassing unit before returning back to the header tank via a filtration system (see [5] for aquarium details).

#### RNA extraction and high-throughput sequencing

**Fig 1:**
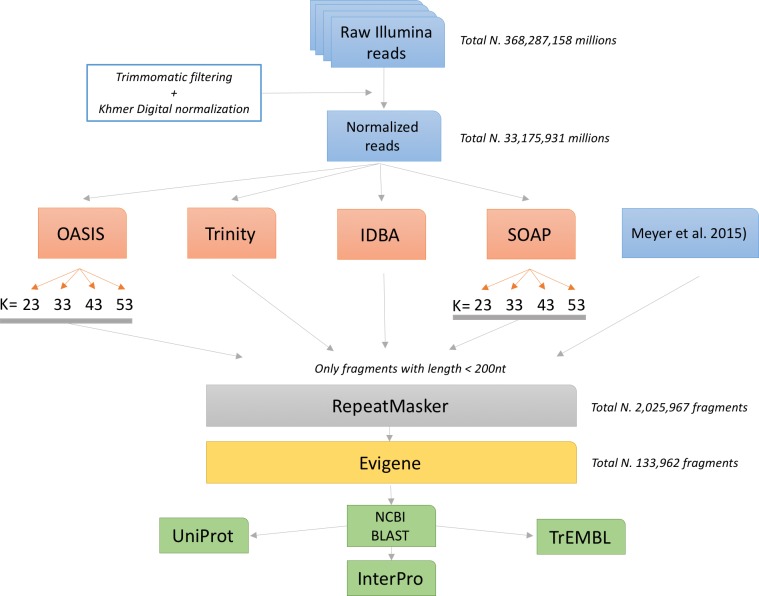
Outline of the assembly process. Raw Illumina reads were first trimmed for adapters and for low quality bases at the 3’ end. They were digitally normalized with the khmer software to reduce redundancy. These sequences were independently assembled using different software (OASIS, Trinity, IDBA and SOAP) and kmer sizes (23, 33, 43, 53). Information deriving from a previous assembly based on the 454 sequencing technology was added to further increase the transcriptome coverage. Repeated sequences were identified and removed using the RepeatMasker software in order to reduce the number of chimeric misassemblies. All surviving fragments were merged into a single transcriptome using the Evidential Gene pipeline. Results were annotated using sequence homology (BLAST) and protein domain searches (InterproScan).

Larvae were transferred from the sea water treatment jars directly into RNAlater® solution using Pasteur pipettes. Total RNA was extracted using the ZR-Duet™ RNA miniPrep procedure (Zymo Research Corporation). Separate extractions were done for each *Furcilia* larva; these were minced with a scalpel blade and briefly homogenized with a microblender in lysis buffer during the first step of RNA extraction. Due to their small size *Calyptopis* larvae were pooled and one RNA extraction performed for each CO_2_ treatment. These samples were homogenised by repeated pipetting in lysis buffer. Quantity and quality of extracted RNA was assessed using a Qubit® 2.0 Fluorometer (Life Technologies) and the Agilent 2100 bioanalyser (Agilent). For Furcilia larvae, RNA extracted from the five individuals in each treatment was pooled in equimolar amounts. Six samples corresponding to the experimental jars (2 larval stages x 3 *p*CO_2_ levels) were sent to GeneWorks (Australia) for sequencing (Fig 1). GeneWorks used a TruSeq RNA sample prep kit (Illumina) and for each sample produced one lane of paired-end reads (2x100 bp) in a Genome Analyzer IIx sequencer (Illumina).

Raw data are accessible on the National Center for Biotechnology Information Short Read Archive (SRA BioProject ID: PRJNA362526, SRA sequences: …….) at the URL: http://www.ncbi.nlm.nih.gov/bioproject/362526

### Transcriptome assembly strategy

The general scheme of the transcriptome assembly and annotation is reported in Fig 1.

#### Transcriptome assemblies

We used Trimmomatic [22] to remove adapter sequences and other artifacts from the raw Illumina sequences. Reads were trimmed starting from the 3’ end until the reported Q score was higher than 3. All resulting reads shorter than 75 nucleotides were discarded.

We then employed the khmer tool (https://dx.doi.org/10.6084/m9.figshare.878460.v2) to normalize the read coverage (“digital normalization”). As suggested by the software documentation, we also trimmed k-mers appearing less than two times in the entire dataset.

The resulting Illumina sequences were independently assembled using four different algorithms: Oases [23], Trinity [24], IDBA [25] and SOAPdenovo-Trans [26]. Both Oases and SOAPdenovo-Trans depend on the choice of the k-mer size used for the construction of the de-Bruijn graph. We thus performed multiple assemblies for different values of *k* (23, 33, 43 and 53) in order to compare the effects of this choice. In all cases, we discarded assembled fragments shorter that 200 nucleotides.

The presence of repeats in a species transcriptome has been linked to the erroneous generation of chimeric fragments. Those are formed by the transcriptome assembler mistakenly joining parts of different transcripts if they happen to share the same repeated sub‑sequence. In order to reduce the number of these errors, we have filtered all the reconstructed fragments using the RepeatMasker software (RepeatMasker Open-4.0. 2013–2015 http://www.repeatmasker.org; we used the RepeatMasker library version 20150807). This procedure removed 29,645 (18%) of the fragments initially assembled.

#### Merging of multiple assemblies

We used the EvidentialGene pipeline (Gilbert D. 2013 https://f1000research.com/posters/5-1695) to identify redundant fragments and to select sequences with the highest coding potential. Table 1 reports the quality measures for this approach. Both fragment lengths and protein matches against the NR database are significantly increased.

**Table 1:** Quality measures of the three de-novo assembly algorithms.

Software	# Frags	# tot Bases	Avg. Frag. Length	N50	% Matches	# Proteins
IDBA	183,688	78,682,848	428 (± 398)	445	13,06%	15,747
Butterfly	190,588	107,415,431	564 (± 596)	770	12,94%	14,927
Oases (*k* = 33)	237,717	159,270,607	670 (± 742)	1,009	13,79%	14,291
SOAP (*k* = 43)	123,855	54,662,513	441 (± 428)	484	16,88%	14,998
Evigene	133,962	129,183,922	964 (± 840)	1,294	67,27%	27,928

To further improve the quality of the transcriptome reconstruction, we used the same software pipeline to merge the results from a previous study which was based on the 454 sequencing technology (175,570 fragments total; see [11]). This approach provided further confirmation of the assembled sequences for 19% of the transcripts in KrillDB.

#### Assembly comparison

We computed a number of measures to assess the quality of the transcriptomes obtained from different assemblers: i) the total number of fragments, ii) the total number of assembled nucleotides, iii) the average fragment length, iv) the N50 index, v) the proportion of fragments linked to known sequences and vi) the ability to reconstruct (putative) full-length transcripts.

#### Functional annotation

We ran BLAST searches to annotate assembled fragments. We aligned our sequences against the NCBI NR (non-redundant) and UniProtKB/TrEMBL protein databases [27,28], and also against the NCBI NT nucleotide collection (data downloaded on 8/10/2015). Results with expectation values greater than 1e-6 for proteins (*blastx*) or 1e-9 for nucleotides (*blastn*) were discarded. Similarly, we ignored all aligned fragments having less than 50% of sequence identity with their target.

To further extend the annotation coverage, we employed InterproScan [29]. This software combines multiple tools to scan novel sequences in search of known functional domains. It is thus able to predict protein family membership.

### Database design and implementation

The pages of KrillDB are dynamically generated by a Python application based on the Flask framework and are served by the Nginx web server. Data is stored in a PostgreSQL 9.4 database (http://www.postgresql.com); full-text searches are implemented using the pg_trgm module. The sequences of the assembled transcripts are available for download as FASTA files.

## Results

### Krill transcriptome assembly

Using Illumina sequencing, we generated a total of 368 million raw reads: 77, 69 and 67 million respectively for control, 1000 or 2000 μatm *p*CO_2_ in *Calyptopis I* samples and 47, 56 and 53 million for *Furcilia V* samples. After the filtering and cleaning process (see Methods for details), a total of 177 million (96,3%) high-quality reads were further used for the assembly.

Four de-novo assembly algorithms have been used on filtered and normalized (with digital normalization) reads: OASES [23], Trinity [24], IDBA [25] and SOAPdenovo-Trans [26]. Similar to what has been observed previously [30], we noticed that the choice of parameters for the assembly algorithms we used had a rather strong impact on the final results. Moreover, it is now increasingly clear that no best method exists but that different assemblers seemed to be able to capture different sets of true transcripts. We thus decided to combine the results from all methods and choice of parameters in order to obtain a more thorough picture of the krill transcriptome. To this aim we use a recent approach (Gilbert D. 2013 https://f1000research.com/posters/5-1695) called EvidentialGene (hereafter evigene) that uses the results of different assembly algorithms extracting the best of their results. Specifically, evigene clusters fragments and then uses the presence and the length of CDS to select the best fragment for a specific transcript among all the available assemblies. Recent works [30,31] have shown that this approach is able to maximize the diversity of the assembled transcripts and their completeness, while limiting sequence redundancy.

For each assembly and for evigene final results we estimated several quality measures based on sequence features (the total number of fragments, their average length, the distribution of frags lengths, the total number of base pairs covered by the assembly and the N50 index) and functional annotation (proportion of fragments with a Blast hit, unique number of NR hits, and the distribution of the protein coverage). The results are reported in Table 1 and Fig 1. Looking at the result of each single assembler we found that OASES gives the highest number of fragments and then the highest number of total base pairs and N50, followed by Trinity and then IDBA and SOAP. The fragments identified by OASES are longer and match with a higher proportion of known proteins, followed by Trinity and then IDBA. On the other hand, OASES assembly seems to be characterized by a higher redundancy (showing a slightly lower number of recognized proteins) with respect to Trinity, SOAP and IDBA. The presence of a higher redundancy is not necessarily a negative aspect, since it may be due to the ability of identifying different isoforms of the same gene. The strategy adopted by evigene reduces the number of fragments, improving fragment length and annotation (Table 1 and Fig 1). It is interesting to note that evigene selects 133,962 fragments, of which 37% derived from OASES annotation, 27% from Trinity, 7% from IDBA, 10% from SOAP and 19% from the previously published transcriptome, confirming the above observation that OASES was on our data the best single assembly algorithm.

Of the 90,121 (67%) fragments of the evigene assembly that have a BLAST hit, 56,026 (62%) have associated at least one GO category. The InterProScan annotation gave us functional annotations for 1,300 other fragments which lacked any BLAST hit.

### Home page of KrillDB

**Fig 2:**
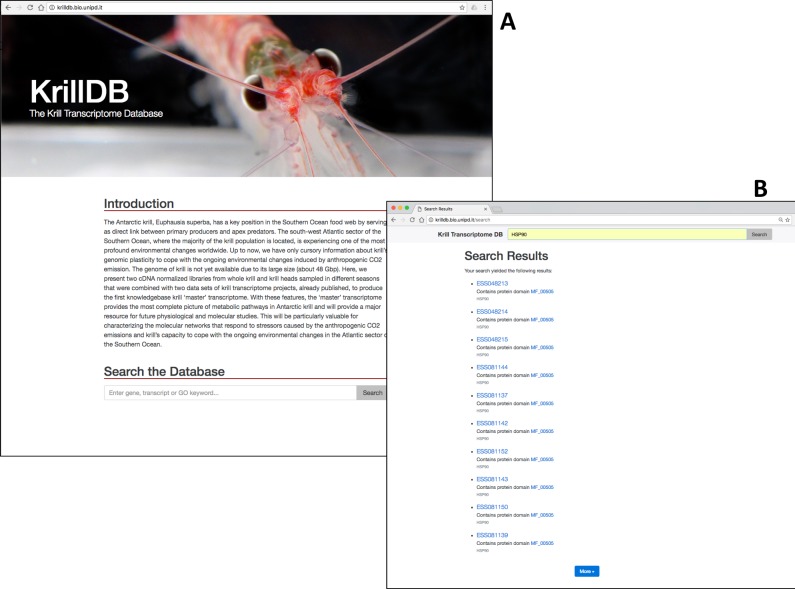
**(A)** The home page of KrillDB. (**B)** An example of search results. Here we queried the database for all hits containing annotations related to the ‘HSP90’ protein family.

Users can access KrillDB at http://krilldb.bio.unipd.it/ (Fig 2A). The home page contains an introduction to KillDB and PolarTime project and gives access to the full text search engine (Fig 2A). The user is free to search any keyword regarding for example the description of the transcript, the Gene Ontology category associated or the protein domain identified.

### Browsing the KrillDB

**Fig 3:**
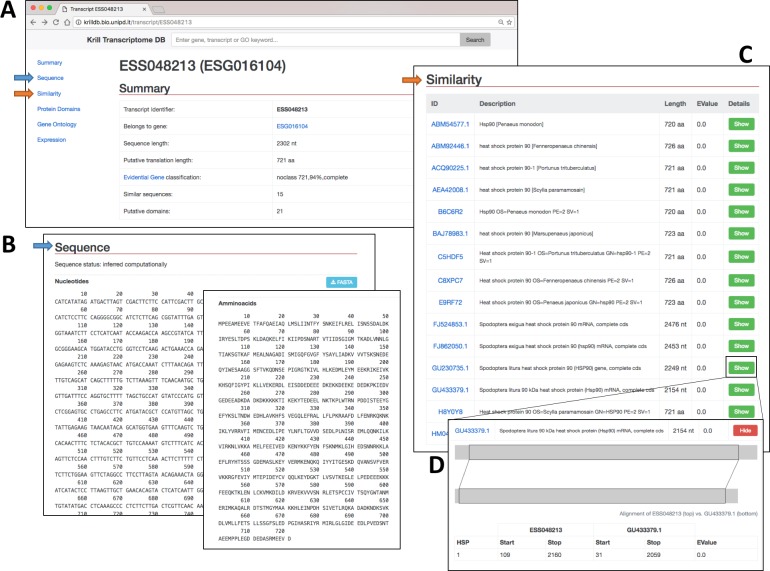
Further exploration of results obtained with the search shown in Fig 2. (A) The summary page for a single transcript. Links to the sequence and similarity sections are highlighted. (**B)** Sequence records. Nucleotide and amino acid sequences are displayed and can be downloaded as text file in the FASTA format. (**C)** Sequence similarity results obtained from BLAST are both summarized in a table and **(D)** depicted graphically to show the matches among different regions of the query and the target sequences.

**Fig 4:**
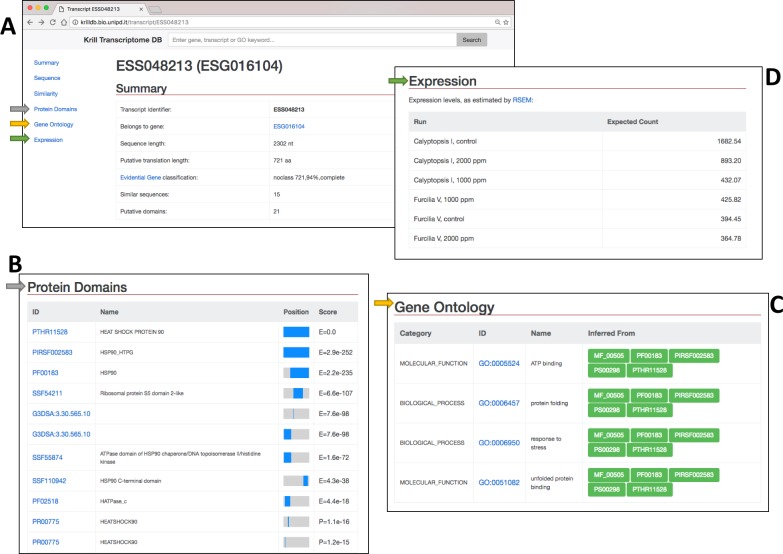
**(A)** Each transcript summary links to detailed sections about protein domains, gene ontology annotations and expression levels. (**B)** Protein domains detected within the transcript are visualized along with their ID, description, e-value and position on the sequence. (**C)** The list of Gene Ontology categories inferred by InterproScan. (**D)** Expression levels for each sequenced sample estimated by the RSEM software.

**Fig 5:**
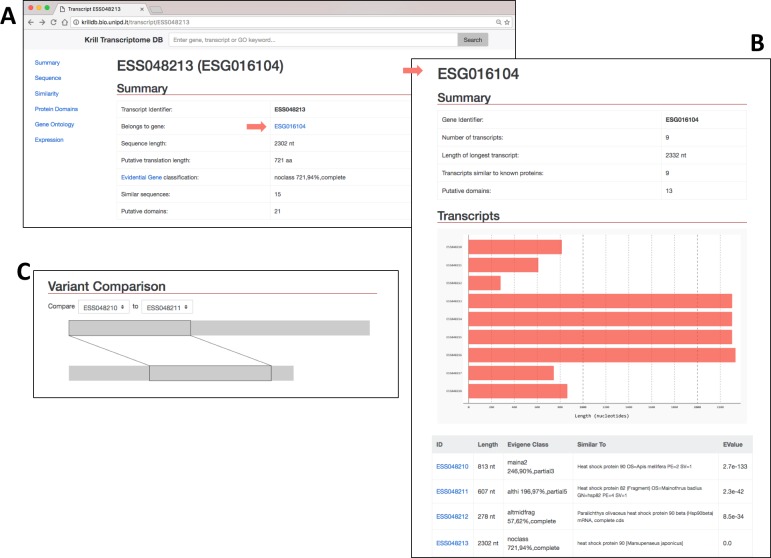
**(A)** Transcript fragments are clustered into groups putatively corresponding to genes. Each transcript page is thus linked to a group page. (**B**)Summary of a transcript group, showing a graphical comparison of the lengths of its members, the most significant BLAST hits and (**C)** pairwise alignment of all transcripts within the group.

The search engine shows the resulted hits into a dynamic web page (Fig 2B). Every hit has a hyperlink that redirects the user to the detailed description the feature. An initial summary is reported with the name of the transcript, the reference gene, the length of the sequence and of the protein, the number of similar sequences (with BLAST) and the number of putative domain identified (Figs 3A, 4A and 5A).

Transcript and protein sequences can be visualized and downloaded as FASTA files (Fig 3B), while in the sequence similarity section (Fig 3C) the ID, the description, the length and the hit e-value are reported. Moreover, for each similar sequence the alignment is visualized and reported as start and end position of the conserved regions (Fig 3D).

In the protein domain section (Fig 4B) the ID (with the link to the reference database), the name, the e-value and the domain position within the protein sequence are reported. In case Gene Ontology categories have been associated to the transcript a dedicated section with the GO ID, the name of the term and the name of the protein domain from which the term definition derived is reported (Fig 4C). If the user selects the name of a protein domain, the GO terms associated with it will be highlighted the above table.

Finally, the expression quantification obtained with RSEM [32] is shown at the end of the page for each of the six libraries (Fig 4D).

Evigene assembly groups reconstructed transcripts according to their similarities. In the summary the ID of the gene (group of transcripts) is reported along with its hyperlink. The web page of the gene (Fig 5B) summarizes key information regarding number of transcripts, their lengths, their annotations along with a visual representation of their pairwise alignments (Fig 5C).

## Conclusions

Anctartic krill are incredibly abundant and represent key species of most food webs in the Southern Ocean. However, the lack of available genome sequences and the limited EST sequences stored in NCBI considerably restrict study progress on the molecular mechanisms of this species. In this study, we aim to produce large numbers of transcript sequences with corresponding annotation information and make these data freely accessible to users. As such, we developed KrillDB, which exhibits simplicity of use for researchers and contains 133,962 transcribed sequences assembled from the combined new Illumina sequences and previously published 454 assembled pyrosequencing data. This database provides information regarding the sequences and functional annotations of the transcripts. The database also includes the developmental stages expression feature for each transcript, which was quantified on RNA-seq data. This database will represent a useful bioinformatics tool for studying molecular processes in krill. KrillDB represents the first large-scale attempt at classifying and annotating the full krill transcriptome. For this reason, we believe it will constitute a cornerstone of future approaches devoted to the study of this species.
